# Are fluent letter dyads really fluent? An update on objective and subjective motor fluency in an Italian student population

**DOI:** 10.1186/s41235-025-00651-4

**Published:** 2025-07-15

**Authors:** Mara Stockner, Giuliana Mazzoni, Francesco Ianì

**Affiliations:** 1 Department of Dynamic and Clinical Psychology, and Health Studies, Sapienza University of Rome, Via Degli Apuli 1, 00185 Rome, Italy; 2 Department of Psychology, University of Turin, Turin, Italy; 3 Centro di Logica, Linguaggio, e Cognizione, University of Turin, Turin, Italy

**Keywords:** Motor fluency, Fluent dyads, Typing, Keyboard

## Abstract

“Motor fluency” refers to the ease with which an action can be performed and several studies have shown how it can modulate various cognitive processes, such as memory and decision making. To investigate these implications of motor fluency, typing-based paradigms have been proven to be useful. In this literature, based on pioneering works that analysed inter-keystroke intervals (IKIs, the time that elapses between two keystrokes), several studies have assumed that letter dyads typed with different hands are more fluent than dyads typed with the same hand. However, to date, there is no literature analysing subjectively perceived typing fluency, i.e. the feeling of fluency experienced by typists. Moreover, this classical conceptualization has not been updated in the last decade. This raises the question of whether this distinction is also reflected in the subjective feeling of fluency, and whether it is still valid in today’s generation of everyday typists. Thus, we investigated the validity of dyad fluency classification by measuring both objective and subjective typing fluency in two samples of university students. The objective measure included both the response times required to type the entire dyads (Experiment 1) as well as reaction times from stimulus presentation to first keypress alongside IKIs (Experiment 2). Overall, we found consistent results that both objective and subjective measures follow the opposite trend compared to classical assumptions: same-hand dyads are (perceived) more fluent than different-hands dyads. Our results have important methodological implications for future research on typing-related motor fluency.

## Introduction

Fluency can be described as the ease with which an internal or external stimulus is processed (e.g., Milhau et al., 2015; Yang et al., 2009). Literature has highlighted different types of fluency such as “perceptual fluency”, i.e. the feeling of ease of visual perception (i.e., Reber et al., 2004) or conceptual fluency related to the comprehension of concepts (e.g., Whittlesea, 1993). Another type of fluency is the so-called “motor fluency” that refers to the (perceived) ease with which an action or series of actions can be performed (e.g., Yang et al., 2009). For example, a natural case of motor fluency is associated with handedness (e.g., Casasanto, 2009). We perform actions mainly with our dominant hand, so right-handed people tend to perform actions more fluently with their right hand (Milhau et al., 2013). Especially within the literature of embodied cognition, the concept of motor fluency has gained notable attention. In this regard, several studies have investigated how motor fluency can modulate various cognitive processes such as recognition memory (Brouillet et al., 2023; Yang et al., 2009), abstract thoughts (Casasanto & Chrysikou, 2011), decision making (e.g., Chen & Lin, 2021) or affective evaluations (e.g., Hayes et al., 2008). This interest is mainly based on the assumption that motor information can be covertly and implicitly activated during the perception of an object (e.g. Barsalou, 2009; Ellis & Tucker, 2000; Ianì et al., 2023). Thus, the mere perception of an object, under certain circumstances, can trigger motor programs that tell us how difficult it would be to interact with the object. For instance, Ping et al. (2009) demonstrated that participants who were asked to observe a series of objects and make preference judgments about them, tended to prefer objects that are easy to interact with, thereby demonstrating how the motor program associated with the object is able to shape participants’ affective evaluations.

In order to investigate the impact of motor fluency on cognitive processes, paradigms based on typing have been shown to be useful. More specifically, according to the standard keyboard input (touch-typing practice), some letter dyads can be defined as “fluent”, i.e. they are typed with two different hands (e.g., AL) as they contain letters that are, spatially, located on different parts of the keyboard. Whilst other dyads are considered as “non-fluent”, they are rather typed with one single hand (e.g., AS) as they contain letters that are closer (and on the same side) on the keyboard. In this line, Beilock and Holt (2007) have, for instance, shown how motor fluency related to the typing of letter dyads impacts likeability ratings. More specifically, the authors presented what have been commonly defined as either “fluent dyads” and “non-fluent dyads” and asked participants to provide preference ratings. They found that expert typists preferred fluent over non-fluent letter dyads, even if they did not perform the typing action during the experiment (see also Van den Bergh et al., 1990). In the same line, Yang et al. (2009) studied the impact of the dyads’ fluency on recognition memory. The authors showed and argued that in a memory task, due to the subjective sense of familiarity elicited by the sense of motor fluency (e.g., Kelly & Jacoby et al., 1998), more recognition errors (i.e., false memories) were made for fluent compared to non-fluent dyads.

These and other studies in cognitive sciences (see also e.g., Cerni et al., 2016; Liang et al., 2008) are based on the assumption that typing dyads with different hands is easier and faster compared to typing letter dyads with the same hand, thereby considering this aspect as a factor able to modulate the perceived motor fluency. Classical typing literature (e.g., Rumelhart & Norman, 1982) hypothesizes that successive keystrokes, based on associated motor programming and execution processes, overlap during typing. Thus, typing two letters with the same finger or hand is expected to result in higher motor interference (in regard to the preparation for the second keystroke) than typing two letters with different hands (see also Coover, 1923; Gentner, 1983; Salthouse, 1984). These assumptions have been supported with data on typing speed as an objective measure of typing fluency. In this regard, studies have shown that it is faster to type dyads that are situated on two different parts of the keyboard (i.e., typed with two different hands: bimanual) than dyads with letters located on the same part of the keyboard (i.e., typed with the same hand: unimanual) (e.g., Salthouse, 1984; Schmuckler & Bosman, 1997).

Whilst objective fluency has been conceptualized as typing speed (e.g., Inter-Keystroke-Intervals (IKI), that is the time elapsing between two keystrokes, e.g., Pinet et al., 2022), to our knowledge, no literature has investigated subjectively perceived typing fluency in this regard. Literature in other fields of fluency has highlighted the importance and implications of subjectively perceived fluency. Forster et al. (2013), for instance, have compared objective and subjective perceptual fluency and found that subjective, more than objective, perceived perceptual fluency predicted liking. Reber et al. (2004) investigated in their study the relationship between subjective (self-reported measure) and objective (reaction time) perceptual fluency, taking into consideration two stages of objective fluency: detection (visually detecting a stimulus) and identification (identifying the nature of the stimulus). They found that the speed of the two subsequent perceptual stages (i.e., objective perceptual fluency in terms of speed) determined the subjective experience of fluency. The same authors suggest that “*speed of processing at different stages condensed into a unified subjective experience of perceptual fluency*” (p. 47).

Regarding typing-related motor processes, research has suggested the interaction between low-level procedural processes (e.g., finger execution) that are not necessarily conscious and more conscious higher-level processes (e.g., typing monitoring). Some studies have also shown that higher-level processes can be disrupted by redirecting attention (e.g., asking typists to focus their attention to their hands while typing, Tapp & Logan, 2011), or that touch typists have more resources for higher-level processes (e.g., internal models of error prediction, Rieger & Bart, 2016). More recently, Pinet and Nozari (2022) have also shown that typing-related behavior, more specifically, error corrections, is not always associated with conscious awareness. To our knowledge, no research has so far investigated whether motor fluency follows the same conceptual organization of typing consisting in lower level (i.e., procedural processes of objective motor fluency in terms of typing speed) and higher-level processes (i.e., subjective awareness of the objective fluency) and the (potential) link between objective and subjective motor fluency. This would provide an important contribution to typing-related metacognition and awareness.

Not only has the subjective nature of *motor* fluency or its relationship with objective motor fluency never been directly investigated, but the literature regarding the classical conceptualization of fluent and non-fluent dyads has not been updated in the last few years either. This is crucial considering recent literature that has shown that typing performance and thus, also typing-related cognition has developed and changed over the last decades (e.g., see evidence by Pinet et al. (2022) on cognitive processes in university students enabling typing being likely those of experts). Not only the increased keyboard exposure in everyday life but also the use of different types of keyboards (i.e., touchscreens, smartphones, tablets etc.) are likely to have changed typing-related behaviors and potentially, also higher-level cognitive processes associated with typing such as subjective awareness of procedural typing processes. Interestingly, some preliminary studies suggest that this may also have impacted (objective) motor fluency related to typing in terms of typing speed. Cerni et al. (2016), for instance, have shown faster IKIs for words containing more unimanual dyads in non-expert typists whilst results of Feit et al. (2016) do not confirm any difference in IKIs for same-hand vs. different-hands keystrokes in expert (i.e., touch) typists.

This leads to the question whether the classical distinction of fluent (i.e., different-hands) vs. nonfluent (same-hand) dyads is also reflected in subjective measures and whether it is still valid in today’s generation of everyday typists. Our study aimed at filling this gap by directly investigating, in two experiments, the validity of the dyad fluency classification by measuring both objective and, for the first time, subjective typing fluency in a university student population. Subjective measures refer to self-reported feeling of fluency, whereas objective fluency measures included both Response Times needed to type the entire letter dyads starting from stimulus presentation (Experiment 1) and Reaction Times (RT; time from stimulus onset to first keypress) alongside IKIs (Experiment 2).

### Experiment 1

In Experiment 1 we aimed at investigating objective and, for the first time, subjective motor fluency regarding the typing of different-hand (the so-called fluent dyads; Beilock & Holt, 2007; Yang et al., 2009) and same-hand (the so-called non-fluent dyads; Beilock & Holt, 2007; Yang et al., 2009) dyads. In order to investigate the validity of the same-hand vs. different-hand dyad categorization (which both our and classic typing paradigms are based on), we assessed which fingers and hands the participants used when typing letter dyads (finger-use task). By applying binomial tests, we expected to encounter a higher probability (compared to chance level) of participants following classical touch-typing hand use.

Moreover, across two experimental tasks, we measured objective motor fluency by instructing participants to type both categories of letter dyads and by measuring their typing speed. We complemented this with self-report responses regarding the subjectively perceived fluency of the typing actions. Additionally, to control for the specific fingers and hands used to type the dyads, in the second motor fluency task, participants were instructed to type the letter dyads with the fingers with which the letters should be typed according to the touch-type system (see e.g., Logan et al., 2016) from which the distinction between fluent and non-fluent dyads emerged. This manipulation allowed us to investigate both motor fluency effects in the case of spontaneous, natural finger-use of typing (spontaneous-typing fluency task) as well as motor fluency effects linked to the original classification of dyads in terms of fluency (touch-typing fluency task), especially relevant in the case results of our task investigating hand use in dyad typing reveals deviations from classical typing behavior.

We expected subjective and objective motor fluency to show the same effect but remained exploratory in our hypothesis regarding which type of dyad should be considered fluent or not (see e.g., Cerni et al., 2016). The study was approved by the ethics committee of the University of Rome La Sapienza.

## Methods

### Participants

Sample size was determined with a priori power analysis based on the publicly available data of Pinet et al. (2022). More specifically, we computed *f*^2^ for their encountered effect of transition type (i.e., bimanual vs. unimanual) on IKIs (see e.g., Gross & Mueller, 2023). A small to medium effect size of *f*^2^ = 0.05 was obtained. We then used the smpsize_lmm () function of the *sjstats* R package (Lüdecke & Lüdecke, 2019). The power analysis yielded a necessary minimum sample size of 20 participants when including a power of 0.80, a significance level of 0.05, two clusters (Dyad Type) and 16 observations per cluster (i.e., number of trials).

24 healthy Italian University students (21 females) participated in the present study (Age: *M* = 25.21 years, *SD* = 3.67). All participants were users of the Italian QWERTY keyboard and had normal to corrected vision. Informed consent was provided by all participants before participating in the experiment.

As today’s university students have been described as proficient typists (e.g. Pinet et al., 2022), we asked participants to complete a typing task in which they typed an Italian sentence (128 characters including spaces, see Appendix A), used as a measure of typing speed/fluency as in Logan and Zbrodoff (1998), to control for participants’ typing proficiency and potentially exclude participants insufficiently unfamiliar with typing. Response Times were measured across all tasks from trial onset to the last keypress that ended the trial, and WPM (words per minute) as a measure of typing speed, was computed according to the following formula (see e.g., Pinet et al., 2022):

$$Numberof \, characters/\left( {5 \, *Time \, taken \, to \, type \, the \, text \, in \, \min utes} \right)$$

Overall, our participants were characterized by a mean typing speed WPM of 48.26 (*SD* = 15.39; range: 19.75—87.20). This range is in line with a study on very large samples of participants conducted by Dhakal and colleagues (2018), who report an overall mean typing speed of 52 WPM, ranging from around 20 (slow typists) to 90 WPM (fast typists) (see also Adhikary & Vertanen, 2021). Indeed, a Welch modified two-sample t-test does not show a statistical difference in WPM between our sample and the sample by Dhakal et al. (2018): *t*(23.01) = − 1.05,* p* = 0.30. Regarding typing accuracy, the mean error rate (calculated as the number of typed words that contained errors; Ianì et al., 2024) was very low with a mean of 0.42 (*SD* = 1.64).

### Materials

32 dyads of the Italian Alphabet were chosen. As in Beilock and Holt (2007) and Yang et al. (2009), all dyads, if typed with the standard touch-typing technique, would be typed with index and middle fingers on an Italian QWERTY keyboard. Half of the dyads (16 items) are fluent dyads, thus typed with two different fingers of two different hands (different-hand dyads) whereas the other half (16 items) are typed with the same finger of the same hand (same-hand dyads) according to the standard touch-typing system (e.g., Logan et al., 2016).

Applying analogous criteria to Yang et al. (2009), we excluded dyads that have a meaning in the Italian language and that represent abbreviations, in order to exclude possible linguistic effects. Finally, we tested potential differences of the frequencies between different-hand and same-hand dyads in the Italian language by retrieving the dyad frequencies from the Italian phonological lexicon created by Goslin et al. (2014) which provided a freely accessible lexical database with phonological representations for 120,000 Italian word forms. Using data derived from this lexicon, the authors also created a series of derived databases and provided estimates of positional frequencies for Italian phonemes and syllables. We found the frequency of different-hand dyads (*M* = 22,356.19; *SD* = 49,576.44) and same-hand dyads (*M* = 10,289.94; *SD* = 26,429.51) not to differ (*t* (30) = 0.859, *p* = 0.39). This was also supported with an independent Bayesian t-test (BF_01_ = 2.24), providing more evidence in favour of the null hypothesis compared to the alternative hypothesis.

### Procedure

**Fig. 1:**
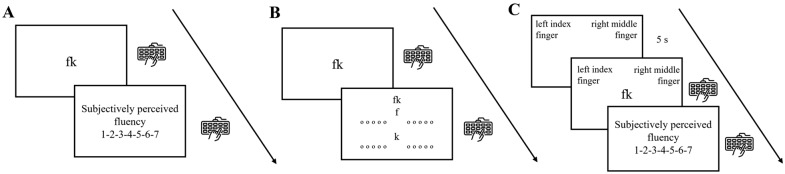
Example trials of Experiment 1 **A** Spontaneous-typing fluency task, **B** Finger-use task and **C** Touch-typing fluency task

The experiment was divided into two experimental motor fluency tasks alongside a task investigating the finger/hand use in typing (i.e., the finger-use task). After signing the informed consent and providing socio-demographic information, the first experimental motor fluency task was administered (spontaneous-typing fluency task). In each trial, one letter dyad was presented in the centre of the screen and participants were instructed to type the dyad. They were instructed to type letter dyads as spontaneously and immediately as possible, but no instructions were given regarding hands/fingers used to type. Subsequently, participants were asked to indicate the perceived fluency of the typing action on a Likert scale ranging from 1 to 7 (1 = not fluent to 7 = fluent), in line with e.g., Forster et al. (2013) (see Fig. 1A). Subsequently, the finger-use task was presented. Participants, first, were presented again with each letter dyad in the centre of the screen and, after having typed the dyad, they indicated with which hands/fingers they would spontaneously type both the first and the second letter of the respective dyad. More specifically, as illustrated in Fig. 1B, the letter dyads remained on the screen and participants indicated, on horizontally aligned finger labels of a single-choice scale, a self-report response regarding their finger use of the first and second letter, respectively.

Then, participants carried out the third experimental task in which they were instructed to type the same letter dyads with the “correct” fingers according to the standard touch-typing system of the QWERTY keyboard (e.g., FK: first letter = left index finger; second letter = right middle finger, see also Fig. 1C and Table 1). More specifically, in each trial, participants saw first a screen with the verbal labels of the indicated finger(s) with the instruction to prepare the finger(s) in order to type the subsequent dyad. After five seconds, the letter dyad appeared on the screen and participants were asked to type the stimulus with the indicated fingers. Subsequently, in line with Task 1, participants were asked to indicate the perceived fluency of the typing action on a Likert scale from 1 to 7 (1 = not fluent to 7 = fluent) (Touch-typing fluency task). In both fluency tasks, all 32 letter dyads were typed and evaluated, and their presentation order was randomized. The whole experiment was carried out on E-prime go.

**Table 1:** Letter dyads and their typing characteristics according to the touch typing criteriatab

Dyad	Type	Finger	Hand
RT	S	II	LL
FR	S	II	LL
FT	S	II	LL
GF	S	II	LL
NM	S	II	RR
UH	S	II	RR
HN	S	II	RR
YU	S	II	RR
GK	D	IM	LR
FK	D	IM	LR
JE	D	IM	RL
MC	D	IM	RL
CY	D	MI	LR
DM	D	MI	LR
IV	D	MI	RL
KR	D	MI	RL
RG	S	II	LL
RV	S	II	LL
VR	S	II	LL
TF	S	II	LL
UM	S	II	RR
HY	S	II	RR
YM	S	II	RR
JN	S	II	RR
RI	D	IM	LR
RK	D	IM	LR
YE	D	IM	RL
YC	D	IM	RL
CN	D	MI	LR
EJ	D	MI	LR
IR	D	MI	RL
IG	D	MI	RL

Type: D = different-hand dyads, S = same-hand dyads; Finger: fingers involved in typing the dyads: I = index finger, M = middle finger; Hands: hands involved in typing the dyads: R = right hand, L = left hand

### Data analysis

All statistical analyses were carried out using RStudio (RStudio Team, 2015, version 2024.04.1). Regarding the finger-use task, a typing score was computed for each trial, attributing 0 to trials in which participants did not follow the expected criteria (i.e., did not type different-hand dyads with two different hands or did not type same-hand dyads with the same hand) and 1 to trials in which participants adopted the expected criteria (i.e., different-hand dyads typed with two different hands and same-hand dyads with the same hand). Then, binomial tests were applied over all trials to test the probability of the participants’ typing scores being greater than chance level (0.50) and thus, indicating them to be more likely to use the expected criteria for typing different-hand and same-hand dyads.

For all models investigating the role of Dyad Type, trials with incorrectly typed dyads were removed (Spontaneous-typing fluency task = 1.69% of trials; Finger-use task: 1.01% of trials; Touch-typing fluency task: 1.43% of trials). Subsequently, outliers (trials) based on typing Response Times were identified by calculating the first (Q1) and third quartile (Q3). Q1—1.5*IQR (interquartile range) presented the lower limit and Q3 + 1.5*IQR the upper limit. Trials below and above these limits were removed (Spontaneous-typing fluency task = 4.77% of all trials; Finger-use task: 5.81%; Touch-typing fluency task: 12.67% of trials).

A mixed effects model approach was adopted for all analyses regarding the role of Dyad Type. In order to investigate the impact of dyad type (i.e., different-hand vs. same-hand dyads, based on touch typing criteria) on objective typing fluency (i.e., Response Times), Linear Mixed Models (LMMs) were run including both Participants as well as Stimuli (i.e., dyads) as random effects. The following model structure was adopted: Response Times ~ Dyad Type + (1 | Participant) + (1|Dyad), implemented with the lmer() function from the *lme4* package (Bates, 2015) and tested with the summary() function.

Moreover, the Likert—scale based responses on subjectively perceived typing fluency were treated as ordinal variables (e.g., Taylor et al., 2023). Thus, ordinal mixed effects regression models were applied implementing the clmm() function from the *ordinal* package (Christensen, 2019). The same model structure as above was applied, including again Participants and Dyads as random effects. The models were tested using the summary() function. Finally, in the touch-typing fluency task, in order to control for potential confounding (interfering or facilitating) lateralization effects (i.e., indicated finger on the left side of the screen could refer to left hand (i.e., congruent condition) but also to right hand (i.e., incongruent hand), see Fig. 1C), Congruence was included as a covariate in the regression models: Dependent Variable ~ Dyad Type + Congruence + (1 | Participant) + (1|Dyad). Congruence was calculated as a trial-by-trial score in which 1 (= congruent) was assigned to trials in which on the left side of the screen a finger of the left hand was presented and on the right hand a finger of the right hand and 0 (= incongruent) in which at least one of the fingers/hands did not correspond to the side of the screen.

Finally, item-by-item Spearman correlations between objective (Response Times) and subjective (Likert-scale responses) fluency measures were computed.

## Results

### Spontaneous-typing fluency task

**Fig. 2:**
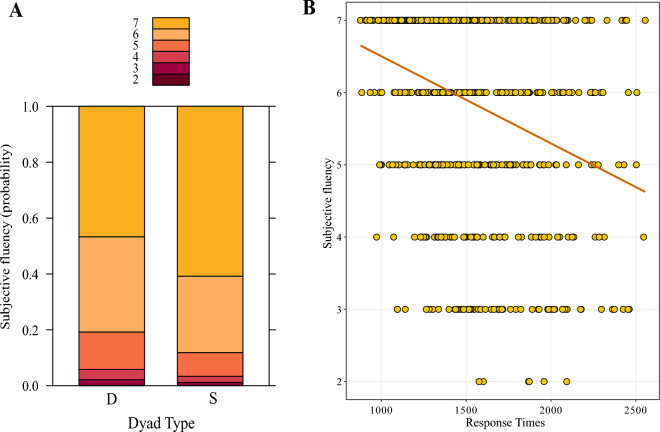
**A** Probability of subjective fluency responses on the Likert scale ranging from 1 (not fluent at all) to 7 (= very fluent), D = Different-hand dyads, S = Same-hand dyads; **B** Correlation between Response Times and subjectively perceived fluency

The LMM on Response Times did not reveal a significant difference in Response Times between different-hand (*M* = 1514.87 ms; *SD* = 354.05) and same-hand letter dyads (*M* = 1.473.61 ms; *SD* = 353.83) (*b* = − 42.42, *SE* = 39.20, *df* = 29.70,* t* = − 1.08, *p* = 0.29). On the other hand, the regression model on subjectively perceived fluency showed a significant difference between different-hand and same-hand dyads (*b* = 0.57, *SE* = 0.24,* z* = 2.37, *p* = 0.017). As illustrated in Fig. 2A, participants were more likely to give higher ratings to same-hand dyads compared to different-hand dyads. Finally, a significant negative correlation between Response Times and subjective fluency was encountered (*ρ* = − 0.34, *p* < 0.0001), see Fig. 2B.

### Finger-use task

In the finger-use task, the probability of success (typing score = 1) for different-hand dyads typing scores was 0.74 whilst probability of success for same-hand dyad typing scores was 0.73. Both binomial tests revealed significant effects (different-hand dyads: 284 out of 384 trials were typed with two different hands, *p* < 0.0001; same-hand dyads: 280 out of 384 trials were typed with the same hand, *p* < 0.0001), indicating that participants, in both types of dyads, tend to adopt the expected typing behavior based on the classification of different-hand and same-hand dyads.^1^ As suggested by the Editor and two anonymous Reviewers, we conducted a small pilot study to test that such a self-report procedure was reliable and thus, that participants’ responses correspond to the objective finger use during typing. More specifically, we have administered the same finger-use task to 10 participants (*M*_*age*_ = 28.70 years, *SD* = 6.72; 7 females) but also filmed their objective typing behavior (i.e., the fingers they used) to control for accuracy. Consistent with the participants in Experiment 1, the participants in our pilot study reported that they followed the classical touch- typing division (i.e., same-hand dyads typed with the same hand and different-hand dyads typed with two different hands) in 74.69% of all trials. Next, we compared the self-reported finger use data with the actual finger use during dyad typing observed in the video recordings, calculating self-report accuracy. We found an overall accuracy of 96.95% in indicating the correct finger for each letter, and the accuracy was comparable between the first letters of the letter dyads (97.48%) and the second letters of the letter dyads (96.21%). In addition, the overall accuracy for letters of different-hand dyads was 98.09% whilst accuracy for same-hand dyads was 95.63%. Overall, these results suggest that participants’ self-report responses regarding their finger use during typing are accurate and that our finger-use task provides reliable results.

Finally, a LMM on Response Times investigating the role of Dyad Type did not reveal a significant difference in Response Times between different-hand (*M* = 1673.26 ms; *SD* = 407.97) and same-hand letter dyads (*M* = 1693.66 ms; *SD* = 440.33) (*b* = 21.24, *SE* = 44.34, *df* = 29.46,* t* = 0.48, *p* = 0.64).

### Touch-typing fluency task

**Fig. 3:**
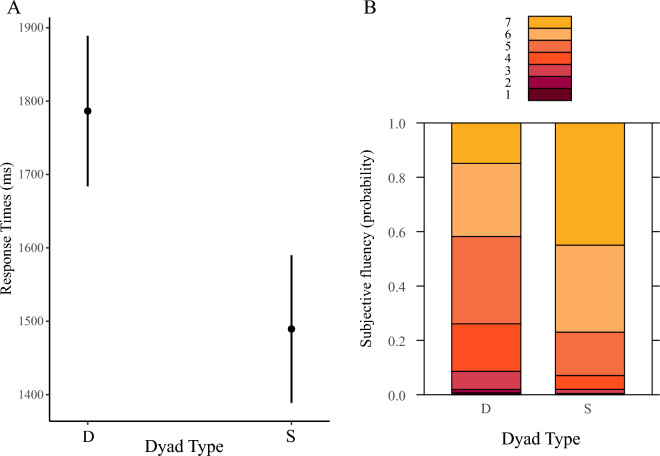
**A** Predicted Response Times (in ms) based on Dyad Type (D = different hand; S = same hand) and **B** Probability of subjective fluency responses on the Likert scale ranging from 1 (not fluent at all) to 7 (= very fluent)

When participants were forced to use the correct fingers according to touch-typing standards, the LMM on Response Times showed a significant effect of Dyad type (*b* = − 370.63, *SE* = 57.20, *df* = 29.40,* t* = − 6.48, *p* < 0.0001). Participants were significantly faster at typing same-hand dyads (*M* = 1481.94; *SD* = 454.62) compared to different-hand dyads (*M* = 1766.31; *SD* = 526.90), see Fig. 3A.

In the same line, Dyad Type was revealed to be a significant predictor of subjective fluency (*b* = 1.88, *SE* = 0.26,* z* = 7.32, *p* < 0.0001). As illustrated in Fig. 3B, same-hand dyads were rated as significantly more fluent compared to different-hand dyads.

Congruence, as a covariate, resulted to be a significant predictor of both Response Times (*b* = − 143.98, *SE* = 66.43, *df* = 30.12,* t* = − 2.17, *p* = 0.04) and subjective fluency (*b* = 0.68, *SE* = 0.28, *z* = 2.39, *p* = 0.02). For both dependent variables, congruent trials led to higher fluency (i.e., higher ratings and faster Response Times).

Finally, as in the spontaneous-typing fluency task, a significant negative correlation between Response Times and subjective fluency was encountered (*ρ* = − 0.31, *p* < 0.0001).

## Discussion

Our results show that in our sample of university students, participants reported they predominantly type letter dyads according to the classical distinction of same-hand vs. different-hand dyads, supporting the persistence of this typing behavior also in today’s generation of everyday typists. Moreover, consistently in both motor fluency tasks, same-hand dyads are subjectively perceived as more fluent. This contrasts with the classical typing literature (e.g., Coover, 1923; Gentner, 1983; Kinkead, 1975) as well as to the existing literature on motor fluency in the field of cognitive psychology that is based on the assumption that same-hand dyads can be considered “nonfluent” dyads (e.g., Beilock & Holt, 2007; Yang et al., 2009). Interestingly, typing speed, measured with Response Times, did not show a significant difference in objective motor fluency in the spontaneous-typing task. However, when participants were forced to use the “correct” fingers, according to the touch-type classification, Response Times showed higher fluency (i.e., were faster) for same-hand dyads. In all tasks and measurements, Response Times correlated negatively with subjective fluency scores, showing that faster typing (lower Response Times) was associated with higher perceived fluency, suggesting how subjective fluency measurements were in line with objective ones.

It is important to note that our measure of typing speed (i.e., from trial onset to the second keystroke) also includes the stages of motor programming and/or preparation before executing the first keystroke. Thus, in order to strengthen our results, we carried out a second study where we measured Inter-Keystroke-Intervals (in line with the typing literature, e.g., Dhakal et al., 2018; Feit et al., 2016; Pinet et al., 2022) and investigated the cognitive implications of objective and subjective motor fluency also assessing dyad likeability.

### Experiment 2

Experiment 1 provided surprising results contrasting the existing literature, our findings suggest that former “non-fluent” (i.e., same-hand) dyads have become more fluent than different-hand dyads. A second experiment confirming this countertrend was necessary to reduce the possibility of a false positive result. Further, as in Experiment 1 we only measured response times, our results are only comparable in a very limited way to classical research in the field of typing where different measures such as reaction times (RT), i.e. the time from stimulus onset to first keypress and Inter-Keystroke-Intervals (IKI), i.e. the time from one keypress to the second keypress, are assessed. Thus, the aim of Experiment 2 was twofold. First, (1) Inter-Keystroke-Intervals (IKIs) were assessed alongside Reaction Times (RT), Response Times and subjectively perceived motor fluency. Second, (2) we decided to further examine the cognitive implications of the subjective (and potentially objective) motor fluency by assessing likeability judgements. As described above, past literature has shown that implicitly activated motor fluency during the observation of motor-related stimuli can modulate affective ratings such as higher likeability of objects that are moved fluently vs. non fluently (e.g., Hayes et al., 2008). Regarding letter dyads, Van den Bergh et al. (1990) as well as Beilock and Holt (2007) have shown that different-hand dyads are preferred over same-hand dyads. In line with our novel results of Experiment 1, also likeability effects due to motor fluency might have changed since the last updates in the literature in this regard. Thus, in order to directly investigate the impact of subjective and objective motor fluency on likeability ratings of letter dyads, Experiment 2 also included a task in which participants were asked to type the letter dyads and subsequently, rate the likeability of each dyad on a Likert scale, allowing for a more insightful understanding of the relationship between motor fluency and degree of likability compared to (previously used) dichotomous Yes–No responses.

We expected to replicate the subjective motor fluency ratings from Experiment 1 (i.e., same-hand dyads to be perceived as more fluent) and that also objective motor fluency in terms of IKIs (as a measure of pure typing speed, excluding other phases of motor programming) or RTs would also follow this pattern. Finally, we expected likeability ratings to be directly related to both types of motor fluency, thus, potentially leading to higher likeability ratings of same-hand dyads. The study was approved by the Ethics committee of the University of Rome La Sapienza.

## Methods

### Participants

Based on the same a-priori power analysis computed for Experiment 1, we recruited 30 healthy Italian University students (26 females) for Experiment 2, their mean age was 26.27 years (*SD* = 10.43). All participants were users of the Italian QWERTY keyboard and had normal to corrected vision. 27 participants were right-handed and 3 participants were left-handed. Informed consent was provided by all participants before participating in the experiment. None of the participants in Experiment 1 were included in Experiment 2.

Overall, in line with the first experiment and Dhakal et al. (2018), participants were characterized by a mean typing speed of 47 WPM (*SD* = 17.50; range: 22.98—96.60). Regarding typing accuracy, the mean error rate (calculated as the number of typed words that contained errors; Ianì et al., 2024) was 0.43 (*SD* = 0.90).

### Materials

The same stimulus set as in the above described Experiment 1 was used.

### Procedure

The experiment consisted of three experimental tasks. In the likeability task, participants were presented with one letter dyad at the time and were asked to type the letter dyad, following the same instructions as the spontaneous-typing fluency task of Exp 1. Subsequently, they were asked to rate on a Likert scale the likeability of each letter dyad (see e.g., Forster et al., 2013; Hayes et al., 2008). More specifically, they were asked to indicate the degree they liked the just typed letter dyad, based on their first impression (in line with Beilock & Holt, 2007), from 1 = not at all to 7 = very much. 

In the second task, in order to investigate the “spontaneous fingers use” of typing and replicate the spontaneous-typing fluency task of Experiment 1, participants then repeated the letter dyad typing task but this time, after having typed each letter dyad, they were asked to indicate the subjectively perceived typing fluency on a Likert scale from 1 = not fluent at all to 7 = very fluent (see Experiment 1, spontaneous-typing fluency task).

Finally, the third task replicated the touch-typing fluency task of Experiment 1 in which participants were instructed to use the “correct” fingers, according to standard touch-typing classification, when typing the letter dyad but also including IKI’s measures. Based on the significant lateralization effects (see Experiment 1, touch-typing fluency task), the layout of the indicated fingers and hands was changed, presenting the finger/hand indicated to type the first letter centered on top of the screen and the finger/hand indicated to type the second letter, underneath, in a second row, again centered on the x-axis. After typing each dyad, participants rated the perceived typing fluency on a Likert scale from 1 (not fluent at all) to 7 (very fluent).

Finally, the same typing task as in Experiment 1 was administered to assess overall typing speed (see above).

Likeability was assessed in the first task in order to prevent the assessment of fluency to influence the assessment of likeability, as the theoretical conceptualization of motor fluency effects suggests that it is the (implicitly) perceived fluency that, consequently, impacts likeability (or affective) judgements (e.g., Hayes et al., 2008; Yang et al., 2009). Therefore, we aimed to avoid activating the explicit metacognitive process of evaluating fluency before assessing likeability.

Inter-Keystroke-Intervals (IKI) were measured by recording the timestamps of the first and second keystroke for each correctly typed dyad. Response Times were again measured across all tasks from trial onset to the last keypress that ended the trial whilst Reaction Times (RT) referred to the time from trial onset to the first keypress. The whole experiment was carried out on E-prime go.

### Data analysis

All statistical analyses were carried out using RStudio (RStudio Team, 2015, version 2024.04.1). As in Experiment 1, trials with incorrectly typed dyads were removed (Likeability task = 2.19% of trials; Spontaneous-typing fluency task: 1.98% of trials; Touch-typing fluency task: 1.15% of trials). Subsequently, outliers based on IKI (as the main variable of interest) were identified by calculating the first (Q1) and third quartile (Q3). Q1—1.5*IQR (interquartile range) presented the lower limit and Q3 + 1.5*IQR the upper limit. Trials below and above these limits were removed (Likeability task = 4.90% of trials; Spontaneous-typing fluency task: 7.12% of trials; Touch-typing fluency task: 7.80% of trials).

A mixed effects model approach was again adopted for all analyses. In order to investigate the impact of dyad type on objective typing fluency (i.e., IKI, RT and Response Times), Linear Mixed Models (LMMs) were run including both Participants as well as Stimuli (i.e., dyads) as random effects. The following model structure was adopted: DV ~ Dyad Type + (1 | Participant) + (1|Dyad), implemented with the lmer() function from the *lme4* package (Bates et al., 2015) and tested with the summary() function. In the case of lacking model fit in the touch-typing fluency task, only Participants were included as random effects.

Moreover, both the Likert—scale based responses on likeability as well as subjectively perceived typing fluency were treated as ordinal variables. Thus, ordinal mixed effects regression models were applied implementing the clmm() function from the *ordinal* package (Christensen, 2019). The same model structure as above was applied, including again Participants and Dyads as random effects. The models were tested using the summary() function. Finally, item-by-item Spearman correlations between objective (Response Times, RT, IKIs) and subjective (likeability and perceived fluency) measures were computed.

## Results

### Likeability task

Regarding objective fluency, the first LMM on Response Times did not reveal a significant effect of Dyad Type (*b* = 10.49, *SE* = 72.17, *df* = 31.64,* t* = 0.15, *p* = 0.86) and neither did RTs (*b* = 43.42, *SE* = 71.40, *df* = 31.89,* t* = 0.61, *p* = 0.55). However, analysis on IKI showed Dyad Type to be a significant predictor (*b* = − 33.63, *SE* = 13.97, *df* = 30.19,* t* = − 2.41, *p* = 0.022). IKIs for same-hand dyads were significantly faster (*M* = 238.69 ms; *SD* = 127.95) compared to different-hand dyads (*M* = 268.11 ms; *SD* = 166.20). The CLMM on likeability ratings did not reveal a significant effect of Dyad Type (*b* = 0.02, *SE* = 0.24, *z* = 0.08, *p* = 0.94).

Finally, a significant negative correlation between likeability and Response Times (*ρ* = − 0.08, *p* < 0.01) but not IKI (*ρ* = − 0.05, *p* = 0.11) or RTs (*ρ* = − 0.06, *p* = 0.053) was found.

### Spontaneous-typing fluency task

**Fig. 4:**
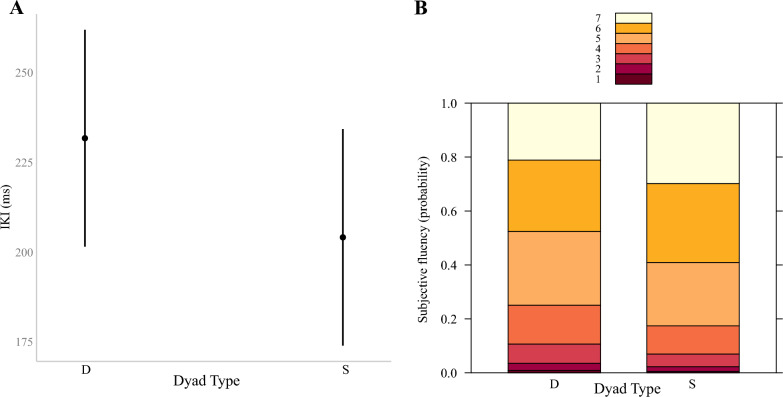
**A** Predicted Inter-Keystroke-Intervals (IKI) in ms based on Dyad Type (D = different-hand; S = same-hand) and **B** Probability of subjective fluency responses on the Likert scale ranging from 1 (not fluent at all) to 7 (= very fluent)

In line with the likeability task, Dyad Type did not significantly predict Response Times (*b* = − 124.31, *SE* = 74.85, *df* = 29.41,* t* = − 1.66, *p* = 0.11) or RTs (*b* = − 97.26, *SE* = 71.80, *df* = 29.22,* t* = − 1.36, *p* = 0.19) but IKIs (*b* = − 27.59, *SE* = 10.23, *df* = 30.42,* t* = − 2.70, *p* = 0.011). More specifically, IKIs of same-hand dyads were again significantly faster (*M* = 199.74 ms, *SD* = 95.65) compared to the IKIs of different-hand dyads (*M* = 225.71 ms; *SD* = 134.64), see also Fig. 4A. Regarding subjectively perceived typing fluency, the CLMM revealed that same-hand dyads were rated as more fluent compared to different-hand dyads (see Fig. 4B), (*b* = 0.46, *SE* = 0.20, *z* = 2.27, *p* = 0.023). Finally, significant negative correlations between subjective fluency and Response Times (*ρ* = − 0.28, *p* < 0.0001) as well as with IKIs (*ρ* = − 0.26, *p* < 0.0001) and with RTs (*ρ* = − 0.22, *p* < 0.0001) were found. A Fisher z-test showed the correlations not to be significantly different (*z* = − 0.53, *p* = 0.59).

### Touch-typing fluency task

**Fig. 5:**
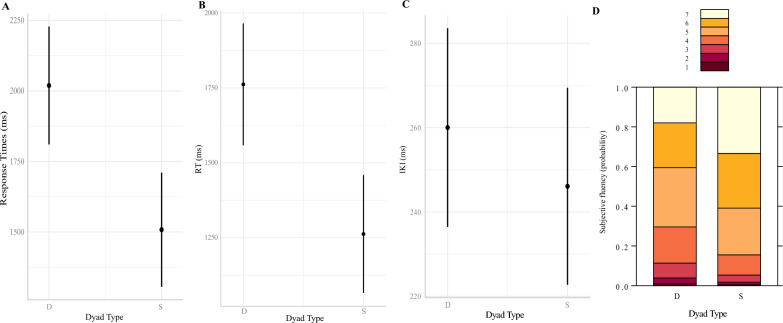
Predicted **A** Response Times, **B** Reaction Times (RT) and **C** Interkey-Keystroke-Intervals (IKI) in ms based on Dyad Type (D = different-hand; S = same-hand) and D) Probability of subjective fluency responses on the Likert scale ranging from 1 (not fluent at all) to 7 (= very fluent)

Regarding objective fluency, the LMM on Response Times revealed a significant effect of Dyad Type (*b* = − 511.45, *SE* = 120.09, *df* = 848.53,* t* = − 4.259, *p* < 0.0001) alongside the LMM on IKIs (*b* = − 13.92, *SE* = 6.83, *df* = 844.32,* t* = − 2.04, *p* = 0.042) and on RTs (*b* = − 499.99, *SE* = 119.89, *df* = 848.93,* t* = − 4.166, *p* < 0.0001). For all three dependent variables, same-hand dyads were characterized by lower values (see Fig. 5ABC).

Dyad Type was also a significant predictor of subjective fluency (*b* = 0.83, *SE* = 0.13, *z* = 6.49, *p* < 0.0001). As illustrated in Fig. 5D, same-hand dyads were more likely to be rated with higher fluency ratings. Finally, significant negative correlations between subjective fluency and Response Times (*ρ* = − 0.23, *p* < 0.0001) as well as IKIs (*ρ* = − 0.14, *p* < 0.0001) and RTs (*ρ* = − 0.21, *p* < 0.0001) were encountered. A Fisher z-test showed the correlations between Response Times and IKIs to be significantly different (*z* = − 2.01, *p* = 0.044), the remaining correlations were statistically comparable (*p*s > 0.09).

## Discussion

Results of Experiment 2 consistently confirm higher subjectively perceived fluency for same-hand compared to different-hand dyads. Further, results on IKI also suggest objective fluency to be greater for same-hand dyads. In line with Experiment 1, when participants were asked to use the correct fingers, Response Times and RTs (along subjective fluency) also showed higher motor fluency for same-hand dyads. Further and unexpectedly, we did not encounter a significant difference in likeability ratings of the different dyad types. Finally, in all tasks and measurements, objective fluency correlated negatively with fluency scores. This means that the faster the typing (i.e., lower RTs, Response Times or IKI), the higher the perceived fluency.

### The role of typing proficiency in motor fluency

Overall, results of both experiments suggest that the classical assumption of many studies (e.g., Beilock & Holt, 2007; Yang et al., 2009) according to which different-hand dyads are perceived as more fluent and typed faster than same-hand dyads may not be robust in today’s generation of everyday typists. Rather, the opposite seems to be the case.

However, it is crucial to consider that motor fluency effects are generally based on motor proficiency (i.e., in our case typing proficiency). Several studies in the literature on typing-related motor fluency reports results based on the typing proficiency of participants (e.g., touch type vs. no-touch type typists, Cerni et al., 2016; Feit et al., 2016 or fast vs. slow typists; e.g., Dhakal et al., 2018, or expert vs. non-expert typists; Beilock & Holt, 2007; Yang et al., 2009). These studies report, throughout different fields of research and consistently, that the fluency assumption of different-hand dyads can be attributed to expert typist (also conceptualized as proficient or touch typists) whilst non-expert typists do not exhibit the same fluency pattern (however, see Pinet et al., 2022 for contrasting results). It could therefore be argued that proficiency level could play a role in our results. First, it is worth noting that our sample included both proficient (i.e., fast) and non-proficient (i.e., slow) typists (both mean and distribution resembled those of the Dhakal et al., 2018 study in which 168,000 participants were tested, see Participants sections’ for more details). Second, in order to be able to compare our (surprising) results with the existing literature, we also considered the role of typing proficiency.

To explore this potential role of typing proficiency in objective and subjective motor fluency findings, we conceptualized typing proficiency as typing speed (i.e., WPM; in line with recent literature showing that typing speed can be considered an indicator of typing proficiency, e.g., Pinet et al., 2022) and carried out exploratory analyses. For all models in which Dyad Type was a significant predictor, we added WPM as a predicting variable in the regression model, allowing it to interact with Dyad Type. To the aim of this section, we discuss only significant interactions between these two factors. In case of interaction, we explored further by applying a median split to our sample, dividing the sample into a proficient (WPM > median) and non-proficient group (WPM < median) and performing the same model on the two subgroups. More specifically the mean WPM of proficient participants was 59.71 (*SD* = 9.26) (Experiment 1) and 59.59 (*SD* = 15.07) (Experiment 2). Participants in the non-proficient groups had a mean WPM of 36.80 (*SD* = 10.29) (Experiment 1) and 33.91 (*SD* = 5.99) (Experiment 2). This can be considered in line with typing speed of previous studies on expert and non-expert typists (e.g., Beilock & Holt, 2007; Cerni et al., 2016; Liang et al., 2008; Yang et al., 2009).

It is worth noting that in the subsequently presented models, the significant main effects of Dyad Type detected in the main analyses of Experiment 1 and 2 were still significant (and are subsequently not discussed again).

*Experiment 1* No significant interaction between Dyad Type and WPM on Response Times was found for the touch-typing fluency task (*b* = 0.37, *SE* = 2.32, *df* = 608.95,* t* = 0.16, *p* = 0.87).

**Fig. 6:**
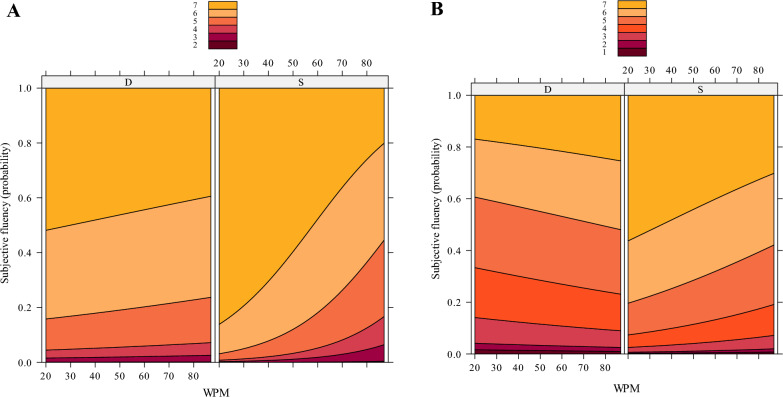
Probability of subjective fluency ratings predicted by WPM and Dyad Type in Experiment 1 in **A** spontaneous-typing fluency task and **B** touch-typing fluency task

Regarding subjective fluency, the interaction between Dyad Type and WPM was significant both for the spontaneous-typing fluency task (*b* = − 0.04, *SE* = 0.01, *z* = − 3.56, *p* = 0.0004) and the touch-typing fluency task (*b* = − 0.02, *SE* = 0.01, *z* = − 2.57, *p* = 0.01). In the spontaneous-typing fluency task, Dyad Type was not significant in proficient typists (*p* = 0.21) but significantly higher fluency for same-hand dyads (compared to different-hand dyads) was encountered in non-proficient typists (*p* = 0.0049), see also Fig. 6A. In the touch-typing fluency task, results show same-hand dyads to be significantly more fluent compared to different-hand dyads both in proficient (*p* < 0.0001) and non-proficient typists (*p* < 0.0001). As illustrated in Fig. 6B, the effect is stronger with decreasing typing proficiency (WPM).^2^

*Experiment 2* WPM did not significantly interact with Dyad Type in predicting Response Times (*p* = 0.22) or RTs (*p* = 0.28) in the touch-typing fluency task.

On IKIs, Dyad Type and WPM interacted significantly in the spontaneous-typing fluency task (*b* = 1.05, *SE* = 0.35, *df* = 812.86, *t* = 3.03, *p* = 0.003) and the touch-typing fluency task (*b* = 1.19, *SE* = 0.38, *df* = 843.13, *t* = 3.05, *p* = 0.002) but not the likeability task (*p* = 0.22). Interestingly, in both the spontaneous-typing and the touch-typing fluency tasks, IKIs were faster for same-hand dyads compared to different-hand dyads in non-proficient typists (spontaneous-typing fluency task: *p* = 0.009, touch-typing fluency task: *p* = 0.005) but not significantly different in proficient typists (spontaneous-typing fluency task: *p* = 0.118; touch-typing fluency task: *p* = 0.899).

**Fig. 7:**
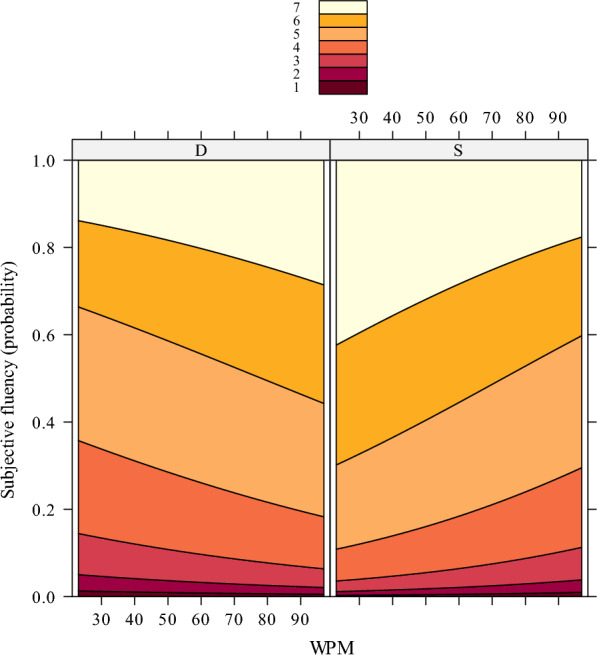
Subjective fluency in the forced touch-typing fluency task (Experiment 2)

Finally, a significant interaction between WPM and subjective fluency was encountered in the touch-typing fluency task (*b* = − 0.03, *SE* = 0.008, *z* = − 3.82, *p* = 0.0001) but not in the spontaneous-typing fluency task (*p* = 0.065). Post hoc comparisons on the touch-typing fluency task illustrated same-hand dyads (vs. different-hand dyads) to be perceived as more fluent in proficient (*p* < 0.0001) and in non-proficient typists (*p* = 0.016). As illustrated in Figure 7, and in line with the touch-typing fluency task of Experiment 1, the effect increases with decreasing typing speed (i.e., WPM).^3^

Overall, results on the role of typing speed provide inconsistent results (see also Table 2). On the one hand, it is important to emphasise that WPM does not seem to play an overall explanatory role in our results: level of proficiency does not always interact with the type of dyad: out of 10 interactions, 5 were not significant. More specifically, it was never relevant for Response Times (Experiment 1, touch-typing fluency task; Experiment 2; touch-typing fluency task) or RTs (Experiment 2, touch-typing fluency task) and did not interact with Dyad Type in predicting IKI in the likeability task (Experiment 2) or subjective fluency in the spontaneous-typing fluency task (Experiment 2).

**Table 2:** Overview of the significant role of WPM for motor fluency based on Dyad Type

Task	Objective fluency			Subjective fluency
	Response times	IKI	RT	
Experiment 1: spontaneous	/	/	/	✔
Experiment 1: touch-type	X	/	/	✔ ✔
Experiment 2: likeability	/	X	/	/
Experiment 2: spontaneous	/	✔	/	X
Experiment 2: touch-type	X	✔	X	✔✔

*/* = not tested because Dyad Type was not significant in main analyses or unavailability of measure; **X** = WPM does not significantly interact with Dyad Type; ✔ WPM does significantly interact with Dyad Type and post-hoc show that effect persists in non-proficient typists; ✔✔ WPM does significantly interact with Dyad Type and post-hoc show that effect persists in both non-proficient typists and proficient typists

On the other hand, it is also important to note that in cases of significant interactions, a closer look reveals a complexity that goes beyond what the literature typically presents. In two cases (subjective fluency in touch-typing fluency task, Experiment 1 and subjective fluency of touch-typing fluency task, Experiment 2), the interaction showed that higher fluency of same-hand dyads compared to different-hand dyads persists in both proficient and non-proficient participants. In the other cases (subjective fluency of spontaneous-typing fluency task, Experiment 1; IKI of spontaneous and touch-typing fluency task, Experiment 2), the interaction revealed higher fluency for same-hand dyads persisted in non-proficient participants whereas it disappeared for proficient participants, a pattern of results not in line with the classical effect assumed by the literature. It is also worth noting that typing speed of our proficient and non-proficient participants is in line with previous studies on expert vs. non-expert typists (i.e., Beilock & Holt, 2007; Cerni et al., 2016; Liang et al., 2008; Yang et al., 2009). In other words, we did not encounter the classical trend (i.e., higher objective fluency/shorter IKIs for different-hand dyads) in any of these tasks or measures.

Overall, our results highlight that the classical distinction between fluent (i.e., different-hand) and non-fluent (same-hand) dyads should not be taken for granted, especially for subjectively perceived fluency (which we have shown to be higher for same-hand dyads in both proficient and non-proficient typists also when WPM is relevant) but also for objective fluency where the same differences seem to disappear in proficient typists but consistently persist in non-proficient typists. In interpreting these results, it is important to consider that our participants included both proficient and less proficient participants with WPM scores largely overlapping with those in literature (e.g., Dhakal et al., 2018).

## General discussion

In two experiments we investigated objective and subjective motor fluency applied to letter dyads and consistently showed how same-hand dyads (in the literature referred to as non-fluent dyads) are perceived as more fluently (Experiment 1 and 2), characterized by faster IKIs (Experiment 2) and also faster Response Times and Reaction Times when participants were instructed to use the fingers according to the touch typing system when typing the dyads (Experiment 1 and 2). These results contrast with the classical literature on typing that was based on the assumption of bimanual dyads being more fluent (e.g., Liang et al., 2008; Rumelhart & Norman, 1982) and suggest that typing-related behavior and cognition have considerably changed in today’s generation of university students. In the literature of the last decade, however, a rather intricate and heterogeneous picture has emerged. For instance, Dhakal et al. (2018) found that bimanual dyads are more fluent (i.e., characterized by faster IKIs) only in fast typists. On the other hand, Feit et al. (2016) has found this to be true only in non-expert (i.e., non-touch type) typists. To our knowledge, there is only one study (i.e., Cerni et al., 2016) that found results in line with our findings. Even though the authors did not directly investigate the fluency of letter dyads (but rather words with more or less bimanual/unimanual transitions), they also found faster IKIs for words with more unimanual transitions. Interestingly, this was only true for non-expert typists whilst the classical pattern (faster IKIs for words with more bimanual transitions) was still found in experts (i.e., touch-typists). The authors did not control for the finger/hand used by the non-experts and thus, hypothesised that their results could be due to the use of unimanual strategies also when typing classical bimanual letter dyads.

In line with this encountered difference based on typing proficiency, it was crucial to further explore the specific role of typing speed for our (unexpected) results. Interestingly, exploratory analyses did not yield consistent results in this regard. First, the level of proficiency (in terms of WPM) interacted with the type of letter manipulation in only 50% of our measurements across all experiments, tasks, and subjective and objective measures. Second and even more importantly, subjective fluency ratings showed higher fluency for same-hand dyads in non-proficient but also proficient typists. For IKIs, on the other hand, higher fluency for same-hand dyads was not observed in proficient typists, but it was confirmed in non-proficient ones. This is, to a certain extent, also in line with Feit et al. (2016) who did not find a significant difference in bimanual vs. unimanual IKIs in more proficient (i.e., touch) typists either. However, their results also suggested that for slow participants, bimanual dyads are considered more fluent compared to unimanual ones. Therefore, whilst these seminal studies partly overlap with our somewhat surprising results, they do not explain the *inversed* effect we encountered (i.e., same-hand dyads being consistently more fluent). Thus, considering the inconsistent role of typing speed for our results, and the typing-related features of our sample (fully in line with the general population as in Dhakal et al., 2018), we argue that our results are not (totally) explainable by the typing proficiency of our sample or predictable based on previous studies. Whilst we found anecdotal evidence that proficiency could play a role for objective measures of fluency (but again not in all cases), our inconsistent findings show little evidence that this might be true for subjective fluency. Overall, the reverse pattern of results (same-hand dyads typed faster and judged to be more fluent than different-hand dyads) seems to be the most robust result of our measurements.

Moreover, our study is, to our knowledge, the first exploration of subjective motor fluency, examining also if it corresponds to objective motor fluency. The subjective component of fluency has been widely discussed in other fields of fluency (e.g., perceptual) regarding the humans’ understanding of the role and function of consciousness (e.g., Forster et al., 2013; Reber, et al., 2004; Topolinski & Strack, 2009). Interestingly, when applying the spontaneous finger-use approach to typing, only IKIs (but not Response Times or RTs) corresponded to the subjectively perceived motor fluency in showing differences between same and different-hand dyads. This may suggest that the consciously perceived component of motor fluency is mainly based on motor *execution* but not motor *planning* processes (which are included in Response Times and RTs but not IKIs). However, this is not fully supported by our overall correlation analysis between objective and subjective fluency. Overall, results showed robust negative associations between objective (Response Times, RTs and IKIs) and subjectively perceived fluency, suggesting that participants are somewhat aware of their objectively measurable typing behavior. Moreover, correlations between IKIs and subjective fluency and Response Times and subjective fluency were either comparable (Exp 2, spontaneous-typing fluency task) or correlations with Response Times were greater compared to the ones with IKI (Exp 2, touch-typing fluency task). The difference between RTs and IKI, on the other hand, did not reach statistical significance (*p* = 0.09). This seems to suggest that motor planning processes (majorly included in Response Times compared to IKIs) also play a crucial role in subjectively perceived fluency, but not only. When comparing these results with the literature on perceptual fluency, it is interesting to note that Reber et al. (2004) hypothesised that objective fluency at different processing stages may condense into one and the same phenomenal experience (i.e., subjective fluency). In contrast, our results suggest that motor processing at different stages might not correlate in the same way to the phenomenal experience of subjective motor fluency. This supports seminal results from Chambon and Haggard (2012) who investigated the impact of fluency on a different metacognitive variable, namely sense of agency. They found that motor *selection* processes but not motor *performance*, contributed to sense of agency, further suggesting that higher-order processes of motor fluency are differentially linked to different motor processes. Whilst our seminal results do not allow for a complete understanding of the components and specific underlying processes of subjective motor fluency yet, they provide a useful starting point for future research investigating the subjective, “consciously perceived” aspects of motor fluency and their implications.

What is the reason for our overall surprising reverse pattern of results? Our research was not designed to identify reasons and possible mechanisms underlying this effect. Therefore, the explanations for our data are purely speculative at the moment, and based on these results future studies need to disentangle the different processes responsible for this aspect. One possible explanation could be that students (like our participants) nowadays do not follow touch-typing rules. However, not only results of our finger-use task suggest that our participants did overall follow the classical distinction of typing different-hand dyads with different hands and same-hand dyads with the same hand; our forced touch-typing fluency tasks also showed consistent effects on same-hand dyads being more fluent, allowing us to rule out that results of our spontaneous-typing fluency tasks are solely due to participants not following traditional touch typing rules. Another relevant aspect could be the (increased) exposure, nowadays, to different types of keyboards. For instance, smartphones and touchscreens, due to different screen sizes and haptics, might not elicit the same classical typing behavior. In this line, studies have shown that typing on smartphones mainly involves the use of the thumbs (e.g., Azenkot & Zhai, 2012; Craighero et al., 2023), and different screens-sizes and haptic characteristics/feedback can modulate typing behavior (e.g., Kim & Than, 2014; Turner et al., 2020). In this regard, for instance, being able to easily and habitually type with one single hand on smartphones and/or tablets (e.g., Bi et al., 2012), might have modulated motor fluency over the last decade. Thus, the increased fluency of same-hand dyads could be the result of an inhibitory process associated with different-hand dyads due to controlled and non-automatized typing gestures (e.g., Tapp & Logan, 2011). However, it is important to highlight that these speculations would need to be empirically tested as, for instance, also smartphones often involve bimanual typing (e.g., Craighero et al., 2023). Future studies could provide important insights, both from a theoretical and empirical point of view, not only for the typing domain but also for other relevant fields of bimanual actions in which bimanual fluency is relevant (e.g., unimanual vs. bimanual coordination, Koeneke et al., 2004).

Finally, we found an overall correlation between IKIs and perceived likeability of letter dyads, suggesting that likeability of action-related stimuli can indeed be associated, at least partially, with the fluency the action is performed (e.g., Hayes et al., 2008). However, the expected implications of distinct motor fluency of same vs. different- hand dyads on likeability ratings yielded null-results. This is not in line with literature suggesting that objective and/or subjectively perceived fluency impacts likeability ratings both in the field of perceptual fluency (e.g., Forster et al., 2013) as well as motor fluency (e.g., Van den Bergh et al., 1990; Beilock & Holt, 2007). It is possible to provide various explanations for this unexpected result. On one hand, literature on motor fluency has demonstrated that likeability judgements are modulated by the sensorimotor simulation of the (typing) action that is activated when evaluating stimuli (e.g., Beilock & Holt, 2007; Hayes et al., 2008). When a secondary motor task involving the same effectors (i.e., fingers) is carried out, this can interfere with the simulation and thus interfere with likeability judgements being impacted by motor fluency (e.g., Beilock & Holt, 2007). In our task, participants both typed the letter dyads and rated likeability via keypresses. This might have interfered with the sensorimotor simulation and might have limited the impact on likeability ratings (instead of oral responses as in Beilock & Holt, 2007 or Hayes et al., 2008). On the other hand, it may also be possible to explain our null-findings with the overall changes in typing-related behaviors over the last decades. Whilst we did find consistent results on objective and subjective motor fluency regarding same-hand dyads, this might not necessarily have implications for affective ratings. Likeability ratings due to (motor) fluency have been shown to be sensitive to a wide range of variables such as specific elements of the stimuli (e.g., necessity of seeing the head and gaze of a person who is performing a fluent vs. non fluent action to be evaluated; Hayes et al., 2008) and not always perceived fluency and liking match (see e.g., Hayes et al., 2008; Topolinski and Strack 2009). Future research could investigate the underlying reasons for the lack of motor fluency impact on likeability when it comes to typing.

Finally, it is worth noting that our study is not without limitations. First, the material we used was intentionally limited to isolated letter dyads which do not frequently occur in Italian. Whilst this allowed us to rule out potential linguistic effects, it would be important to extend our results to other, more ecological, materials (e.g., words or texts) before generalizing them. Moreover, stimuli could also be manipulated based on keyboard characteristics such as distance (and/or rows) between keypresses, as well as including same-finger and different-finger unimanual letter dyads which might elicit different effects (see e.g., Larochelle, 1983). Additionally, whilst our forced touch-typing fluency tasks allowed us to exclude that our results were due to participants not following touch-typing rules, we cannot exclude that forcing participants to type in a specific way might have implicated enhanced monitoring processes or other additional higher-level processes (e.g., Tapp & Logan, 2011). Indeed, exploratory analyses revealed an overall decrease in fluency scores between the spontaneous and the touch-typing fluency task (i.e., slower typing of dyads, lower subjective fluency), thus, results on the touch-typing fluency tasks should be interpreted with caution and seen as preliminary findings in this regard. However, the overall encountered effect between different-hand and same-hand dyads remained consistent. Whilst this allowed us to rule out that our main finding (i.e., same-hand dyads defined by touch-typing criteria are more fluent than different-hand dyads defined by touch-typing criteria) is due to participants not following the traditional touch-typing behavior, replicating our results on touch-typists (vs. non-touch typists) would represent a cleaner method for controlling for this. Finally, regarding our experimental procedure, order of tasks was fixed as we expected practice effects would be very limited given the nature of our stimuli; however, future research could take into consideration potential practice effects and investigate how practicing impacts motor fluency or likeability ratings based on motor fluency. In this line, future studies could also expand the experimental procedure by implementing the finger-use task or video-recordings (as in our pilot study) in the motor fluency tasks measuring IKI and subjective fluency. This would allow categorizing dyads (or other motor-related stimuli) based on the participants’ actual motor behavior (instead of only using literature-based fluency categories) and allow for a more detailed insight and comparison of fluency measures. Moreover, it would also provide insight into typing (in)consistency throughout different tasks, as an index of (fixed) motor schemata usually associated with expert (i.e., touch) typists (e.g., Feit et al., 2016). 

To summarize, the present study provides novel insights into the subjective component of motor fluency as well as an update of the fluency classification regarding letter dyads. This result cautions against the classical assumption that bimanual dyads are more fluent than unimanual ones. Possible explanations and methodological implications should be considered in future studies. 

## Data Availability

All data is available at: https://osf.io/d9ub5/?view_only=e895030bd27f4ec1a8bfdda87e4c3140

## Appendix A

### Text of the typing task.

Poi andarono alla ricreazione ma prima presero tutti i loro panieri con dentro la colazione che erano appesi ai muri dalle ore 5.

## Footnotes

1 Results related to the finger-use task have also been (partially) reported in Stockner et al. (2025), Appendix A.

2 Proficient typists: different-hand dyads (*M* = 5.28, *SD* = 1.66), same-hand dyads (*M* = 5.90, *SD* = 1.29); non-proficient typists (different-hand dyads: *M* = 4.89, *SD* = 1.42; same-hand dyads: *M* = 5.90, *SD* = 1.23).

3 Proficient typists: different-hand dyads (*M* = 5.09, *SD* = 1.60), same-hand dyads (*M* = 5.39, *SD* = 1.48); non-proficient typists (different-hand dyads: *M* = 4.87, *SD* = 1.51; same-hand dyads: *M* = 5.80, *SD* = 1.23).

## References

[CR1] Azenkot, S., & Zhai, S. (2012). Touch behavior with different postures on soft smartphone keyboards. In *Proceedings of the 14th international conference on Human-computer interaction with mobile devices and services* (pp. 251–260).

[CR2] Adhikary, J., & Vertanen, K. (2021). Typing on midair virtual keyboards: exploring visual designs and interaction styles. In *IFIP conference on human-computer interaction* (pp. 132–151). Cham: Springer International Publishing.

[CR4] Barsalou, L. W. (2009). Simulation, situated conceptualization, and prediction. *Philosophical Transactions of the Royal Society b: Biological Sciences,**364*(1521), 1281–1289. 10.1098/rstb.2008.0319 PMC2666716 19528009

[CR5] Bates, D., Maechler, M., Bolker, B., Walker, S., Christensen, R. H. B., Singmann, H., Dai, B., Grothendieck, G., Green, P., & Bolker, M. B. (2015). Package ‘lme4.’ *Convergence,**12*(1), 2.

[CR6] Beilock, S. L., & Holt, L. E. (2007). Embodied preference judgments: Can likeability be driven by the motor system? *Psychological Science,**18*(1), 51–57. 17362378 10.1111/j.1467-9280.2007.01848.x

[CR7] Bi, X., Chelba, C., Ouyang, T., Partridge, K., & Zhai, S. (2012). Bimanual gesture keyboard. In *Proceedings of the 25th annual ACM symposium on User interface software and technology* (pp. 137–146).

[CR8] Brouillet, D., Rousset, S., & Perrin, D. (2023). Experience of memory: Transfer of the motor feeling of fluency linked to our interaction with the environment. *Psychological Research Psychologische Forschung,**87*(6), 1753–1760. 36574018 10.1007/s00426-022-01759-8

[CR9] Casasanto, D. (2009). Embodiment of abstract concepts: Good and bad in right- and left-handers. *Journal of Experimental Psychology: General,**138*(3), 351–367. 19653795 10.1037/a0015854

[CR10] Casasanto, D., & Chrysikou, E. G. (2011). When left is “right” motor fluency shapes abstract concepts. *Psychological Science,**22*(4), 419–422. 21389336 10.1177/0956797611401755

[CR11] Cerni, T., Velay, J. L., Alario, F. X., Vaugoyeau, M., & Longcamp, M. (2016). Motor expertise for typing impacts lexical decision performance. *Trends in Neuroscience and Education,**5*(3), 130–138.

[CR12] Chambon, V., & Haggard, P. (2012). Sense of control depends on fluency of action selection, not motor performance. *Cognition,**125*(3), 441–451. 22902284 10.1016/j.cognition.2012.07.011

[CR13] Chen, M., & Lin, C. H. (2021). What is in your hand influences your purchase intention: Effect of motor fluency on motor simulation. *Current Psychology,**40*(7), 3226–3234.

[CR14] Christensen (2019). Ordinal–Regression models for ordinal data. R package version 2019.12–10. https://CRAN.R-project.org/package=ordinal

[CR15] Coover, J. E. (1923). A method of teaching typewriting based upon a psychological analysis of expert typing. *National Education Association,**61*, 561–567.

[CR16] Craighero, L., Granziol, U., & Sartori, L. (2023). Digital intentions in the fingers: I know what you are doing with your smartphone. *Brain Sciences,**13*(10), 1418. 37891787 10.3390/brainsci13101418 PMC10605869

[CR17] Dhakal, V., Feit, A. M., Kristensson, P. O., & Oulasvirta, A. (2018, April). Observations on typing from 136 million keystrokes. In *Proceedings of the 2018 CHI conference on human factors in computing systems* (pp. 1–12).

[CR18] Ellis, R., & Tucker, M. (2000). Micro-affordance: The potentiation of components of action by seen objects. *British Journal of Psychology,**91*(4), 451–471. 11104173 10.1348/000712600161934

[CR19] Feit, A. M., Weir, D., & Oulasvirta, A. (2016, May). How we type: Movement strategies and performance in everyday typing. In *Proceedings of the 2016 chi conference on human factors in computing systems* (pp. 4262–4273).

[CR20] Forster, M., Leder, H., & Ansorge, U. (2013). It felt fluent, and I liked it: Subjective feeling of fluency rather than objective fluency determines liking. *Emotion,**13*(2), 280. 23088777 10.1037/a0030115

[CR21] Gentner, D. R. (1983). The acquisition of typewriting skill. *Acta Psychologica,**54*(1–3), 233–248.

[CR22] Goslin, J., Galluzzi, C., & Romani, C. (2014). PhonItalia: A phonological lexicon for Italian. *Behavior Research Methods,**46*, 872–886. 24092524 10.3758/s13428-013-0400-8

[CR23] Groß, J., & Möller, A. (2023). Effect Size Estimation in Linear Mixed Models. *arXiv preprint *arXiv:2302.14580.

[CR24] Hayes, A. E., Paul, M. A., Beuger, B., & Tipper, S. P. (2008). Self produced and observed actions influence emotion: The roles of action fluency and eye gaze. *Psychological Research Psychologische Forschung,**72*(4), 461–472. 17899177 10.1007/s00426-007-0125-3

[CR25] Ianì, F., Limata, T., Bucciarelli, M., & Mazzoni, G. (2023). The implicit effect of action mental simulation on action evaluation. *Quarterly Journal of Experimental Psychology,**76*(2), 257–270. 10.1177/17470218221091096 35306935

[CR26] Ianì, F., Stockner, M., & Mazzoni, G. (2024). Explicit and implicit memory for the QWERTY keyboard: The role of motor simulation and deictic gestures. *Attention, Perception, & Psychophysics,**86*(2), 602–615. 10.3758/s13414-023-02829-8 38135782

[CR27] Kelley, C. M., & Jacoby, L. L. (1998). Subjective reports and process dissociation: Fluency, knowing, and feeling. *Acta Psychologica,**98*(2–3), 127–140.

[CR28] Kim, J. R., spsampsps Tan, H. Z. (2014). Haptic feedback intensity affects touch typing performance on a flat keyboard. In *Haptics: Neuroscience, Devices, Modeling, and Applications: 9th International Conference, EuroHaptics 2014, Versailles, France, June 24-26, 2014, Proceedings, Part I 9* (pp. 369-375). Springer Berlin Heidelberg 10.1007/978-3-662-44196-1_44 PMC4331022 25699293

[CR29] Kinkead, R. (1975, October). Typing speed, keying rates, and optimal keyboard layouts. In *Proceedings of the Human Factors Society Annual Meeting* (Vol. 19, No. 2, pp. 159–161). Sage CA: Los Angeles, CA: SAGE Publications.

[CR30] Koeneke, S., Lutz, K., Wüstenberg, T., & Jäncke, L. (2004). Bimanual versus unimanual coordination: What makes the difference? *NeuroImage,**22*(3), 1336–1350. 15219606 10.1016/j.neuroimage.2004.03.012

[CR31] Larochelle, S. (1983). A comparison of skilled and novice performance in discontinuous typing. *Cognitive aspects of skilled typewriting* (pp. 67–94). Springer.

[CR32] Liang, H. W., Hwang, Y. H., & Chang, F. H. (2008). Temporal change in bimanual interkeypress intervals and self-reported symptoms during continuous typing. *Journal of Occupational Rehabilitation,**18*, 319–325. 18830806 10.1007/s10926-008-9150-3

[CR33] Logan, G. D., Ulrich, J. E., & Lindsey, D. R. (2016). Different (key) strokes for different folks: How standard and nonstandard typists balance Fitts’ law and Hick’s law. *Journal of Experimental Psychology: Human Perception and Performance,**42*(12), 2084. 10.1037/xhp0000272 27748613 10.1037/xhp0000272

[CR34] Logan, G. D., & Zbrodoff, N. J. (1998). Stroop-type interference: Congruity effects in color naming with typewritten responses. *Journal of Experimental Psychology: Human Perception and Performance,**24*(3), 978.

[CR35] Lüdecke, D., & Lüdecke, M. D. (2019). Package ‘sjstats’. *Statistical functions for Regression Models, Version 0.17*, *3*.

[CR37] Milhau, A., Brouillet, T., & Brouillet, D. (2013). Biases in evaluation of neutral words due to motor compatibility effect. *Acta psychologica,**144*(2), 243–249. 23920406 10.1016/j.actpsy.2013.06.008

[CR36] Milhau, A., Brouillet, T., & Brouillet, D. (2015). Valence–space compatibility effects depend on situated motor fluency in both right-and left-handers. *Quarterly Journal of Experimental Psychology,**68*(5), 887–899. 10.1080/17470218.2014.967256 25379954

[CR38] Pinet, S., Zielinski, C., Alario, F. X., & Longcamp, M. (2022). Typing expertise in a large student population. *Cognitive Research: Principles and Implications,**7*(1), 77. 35930064 10.1186/s41235-022-00424-3 PMC9356123

[CR39] Ping, R. M., Dhillon, S., & Beilock, S. L. (2009). Reach for what you like: The body’s role in shaping preferences. *Emotion Review,**1*(2), 140–150.

[CR40] Reber, R., Wurtz, P., & Zimmermann, T. D. (2004). Exploring “fringe” consciousness: The subjective experience of perceptual fluency and its objective bases. *Consciousness and Cognition,**13*(1), 47–60. 14990240 10.1016/S1053-8100(03)00049-7

[CR41] Rieger, M., & Bart, V. K. (2016). Typing style and the use of different sources of information during typing: an investigation using self-reports. *Frontiers in psychology,**7*, 1908. 28018256 10.3389/fpsyg.2016.01908 PMC5145878

[CR42] Rumelhart, D. E., & Norman, D. A. (1982). Simulating a skilled typist: A study of skilled cognitive-motor performance. *Cognitive Science,**6*(1), 1–36.

[CR43] RStudio Team. (2015). RStudio: Integrated development for R (Version 2024.4.1.748) [Computer software]. RStudio, Inc. http://www.rstudio.com/

[CR44] Salthouse, T. A. (1984). Effects of age and skill in typing. *Journal of Experimental Psychology: General,**113*(3), 345. 6237168 10.1037//0096-3445.113.3.345

[CR45] Schmuckler, M. A., & Bosman, E. L. (1997). Interkey timing in piano performance and typing. *Canadian Journal of Experimental Psychology/revue Canadienne De Psychologie Expérimentale,**51*(2), 99. 9340078 10.1037/1196-1961.51.2.99

[CR46] Stockner M., Mazzoni, G. & Ianì F (*in press*). Motor fluency and preference judgments: typing speed as a predictor of letter dyads’ likeability.

[CR47] Tapp, K. M., & Logan, G. D. (2011). Attention to the hands disrupts skilled typewriting: The role of vision in producing the disruption. *Attention, Perception, & Psychophysics,**73*(8), 2379–2383. 10.3758/s13414-011-0208-5 21915764

[CR48] Taylor, J. E., Rousselet, G. A., Scheepers, C., & Sereno, S. C. (2023). Rating norms should be calculated from cumulative link mixed effects models. *Behavior Research Methods,**55*(5), 2175–2196. 36103049 10.3758/s13428-022-01814-7 PMC10439063

[CR49] Topolinski, S., & Strack, F. (2009). Scanning the “fringe” of consciousness: What is felt and what is not felt in intuitions about semantic coherence. *Consciousness and Cognition,**18*(3), 608–618. 18650104 10.1016/j.concog.2008.06.002

[CR50] Turner, C. J., Chaparro, B. S., Sogaard, I. M., & He, J. (2020). The effects of keyboard layout and size on smartphone typing performance. In *Proceedings of the Human Factors and Ergonomics Society Annual Meeting* (Vol. 64, No. 1, pp. 985–989). Los Angeles, CA: SAGE Publications.

[CR51] Van den Bergh, O., Vrana, S., & Eelen, P. (1990). Letters from the heart: Affective categorization of letter combinations in typists and nontypists. *Journal of Experimental Psychology: Learning, Memory, and Cognition,**16*(6), 1153.

[CR52] Whittlesea, B. W. (1993). Illusions of familiarity. *Journal of Experimental Psychology: Learning, Memory, and Cognition,**19*(6), 1235.

[CR53] Yang, S. J., Gallo, D. A., & Beilock, S. L. (2009). Embodied memory judgments: A case of motor fluency. *Journal of Experimental Psychology: Learning, Memory, and Cognition,**35*(5), 1359. 19686029 10.1037/a0016547

